# Durable superhydrophobic interfaces sustain long-term ammonia recovery from real anaerobic digestion effluent

**DOI:** 10.1016/j.ese.2026.100748

**Published:** 2026-08-04

**Authors:** Hongrae Im, Am Jang

**Affiliations:** Department of Global Smart City, Sungkyunkwan University (SKKU), 2066, Seobu-ro, Jangan-gu, Suwon, Gyeonggi-do, 16419, Republic of Korea

**Keywords:** Nutrient recovery, Membrane contactor, Superhydrophobic interface, Optical coherence tomography, Anti-wetting

## Abstract

Ammonia recovery from wastewater supports sustainable nitrogen circularity and reduces reliance on energy-intensive Haber-Bosch synthesis. Anaerobic digestion effluent represents a concentrated nitrogen resource, yet membrane contactors for selective NH_3_ recovery suffer from coupled wetting and fouling in complex real matrices, limiting long-term performance. Conventional hydrophobic modifications improve initial resistance but lack quantitative mechanistic understanding of how interface design governs pore-scale dynamics and sustained transport under realistic conditions. Here we show that systematic fluorosilane engineering of commercial PVDF membranes produces superhydrophobic contactors that, when guided by operando optical coherence tomography, establish a quantitative framework linking interfacial fluorination density to the stabilization of Cassie-Baxter-like states, suppression of pore wetting and fouling, and durable high-efficiency NH_3_ recovery from real anaerobic digestion effluent. The optimal PVDF@PFDTMS membrane achieves 98.4% recovery efficiency and 44.8 g m^−2^ d^−1^ flux while maintaining near-invariant porosity and thickness over 20 h and across multiple physical cleaning cycles. These results demonstrate that operando structural diagnostics can transform empirical surface modification into a rational, mechanism-informed design strategy for resilient membrane contactors. This framework advances durable, energy-efficient nutrient recovery technologies for high-strength waste streams and supports scalable nitrogen circularity.

## Highlights

• Optical coherence tomography links fluorinated interfaces to durable performance in real anaerobic digestion effluent.
• Optical coherence tomography-guided interfaces enable sustained ammonia recovery with structural stability.
• Real anaerobic digestion effluent reveals limits of conventional superhydrophobic designs for long-term stability.
• Multi-cycle physical cleaning restores performance of mechanistically designed superhydrophobic membrane contactors.
• This work advances mechanism-based design for durable ammonia recovery from complex wastewater.

## Graphical abstract

**Figure:**
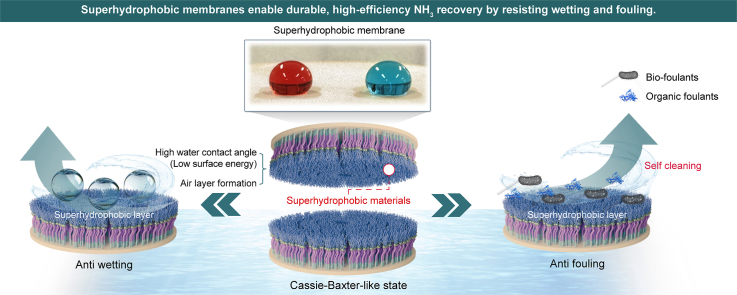

## 1 Introduction

Ammonia (NH_3_) is a foundational chemical underpinning modern agriculture, industry, and emerging energy systems [1]. It serves as the primary precursor for nitrogen fertilizers and is widely used in refrigerants, pharmaceuticals, and specialty chemicals [2]. In addition, NH_3_ has been discussed as a potential carbon-neutral energy carrier when synthesized using low-carbon hydrogen and renewable electricity [3]. Global NH_3_ production exceeds 200 million tons annually and is predominantly made using the Haber-Bosch (H-B) process [4]. However, conventional H-B synthesis is highly energy- and carbon-intensive due to its reliance on fossil-derived hydrogen and operation under elevated temperature and pressure [5]. Current estimates indicate that H-B NH_3_ production consumes approximately 3-5% of the global natural gas supply, requires on the order of 10 kWh per kg N of energy input, and accounts for roughly 1-2% of global CO_2_ emissions [6]. These environmental burdens have intensified interest in strategies to improve nitrogen circularity and reduce dependence on fossil-intensive NH_3_ production routes [7]. Concurrently, NH_3_ discharge from wastewater represents a significant environmental challenge [8]. Elevated nitrogen loading can accelerate eutrophication, stimulate harmful algal blooms, and degrade aquatic ecosystem health [9]. Wastewater, therefore, constitutes both a pollution source and a secondary nitrogen reservoir [10]. Although conventional biological nitrification–denitrification can achieve stringent nitrogen discharge standards, it is energy intensive and ultimately converts reactive nitrogen to N_2_, thereby forfeiting opportunities for nutrient recovery [11]. From a circular water–energy–resource perspective, technologies that enable NH_3_ recovery rather than removal are more aligned with sustainable nitrogen management.

High-strength NH_3_-containing waste streams, including semiconductor etching effluents, source-separated urine, landfill leachate, and livestock digestate, are particularly attractive targets for recovery-oriented treatment due to their elevated nitrogen concentrations [12]. Among these, anaerobic digestion effluent (ADE) is of special relevance because anaerobic digestion (AD) is widely applied to food waste, sewage sludge, and manure stabilization [13]. While AD efficiently converts organic matter into biogas, it conserves nitrogen by transforming organic nitrogen into ammonium (NH_4_^+^) [14]. Consequently, ADE typically contains high total ammonia nitrogen (TAN), with reported concentrations ranging from approximately 600–5900 mg L^−1^ depending on feedstock and operating conditions [15]. Such TAN levels can inhibit nitrifying microorganisms and complicate downstream biological treatment, while uncontrolled discharge contributes to nitrogen loading in the receiving waters [16]. Owing to its relatively high NH_3_ concentration and moderate flow rates compared to mainstream wastewater, ADE represents a technically and economically favorable sidestream for selective NH_3_ recovery technologies [17].

A variety of physicochemical and membrane-based processes have been explored for the treatment and recovery of NH_3_-rich wastewater, including adsorption, air or steam stripping, ion exchange, electrolytic precipitation, bipolar membrane electrodialysis, and pressure-driven membrane technologies [18,19]. However, many of these approaches suffer from distinct operational bottlenecks (Supplementary Table S1); these include competitive ion adsorption, high chemical costs for pH adjustment, electrode passivation, and severe membrane fouling or extreme energy demands in hybrid configurations, such as ultrafiltration or microfiltration coupled with stripping or evaporation [20]. Moreover, membrane performance can be significantly affected by matrix complexity, including high ionic strength and elevated organic content, both of which are characteristic of ADE [21]. These limitations underscore the need for selective, durable, and operationally robust recovery platforms capable of sustained performance under realistic wastewater conditions [22]. In a recovery-oriented framework, NH_3_ can be captured and converted into value-added ammonium salts, such as ammonium sulfate or ammonium nitrate, thereby contributing to nutrient circularity and partially offsetting fossil-derived NH_3_ production [23].

Membrane contactors (MCs) have emerged as a promising platform for the selective recovery of volatile compounds, including NH_3_ [[24], [25], [26]]. Unlike pressure-driven membrane separation, MCs maintain a non-dispersive gas–liquid interface within the pores of a hydrophobic membrane [15]. In NH_3_ recovery configurations, the feed solution is alkalized to shift the NH_4_^+^/NH_3_ equilibrium toward volatile NH_3_, which diffuses through air-filled pores and is absorbed into an acidic stripping solution to form ammonium salts [27]. Owing to their high interfacial area, modular design, and low hydraulic energy demand, MC systems are attractive for decentralized or on-site nutrient recovery [28].

Despite these advantages, long-term MC performance is fundamentally governed by the stability of the gas–liquid interface within the membrane pores [29]. Membrane wetting, defined as liquid intrusion into hydrophobic pores, displaces the gas phase, increases mass-transfer resistance, and can lead to undesired solute crossover [30]. Under wetted conditions, pores may transition from vapor pathways to liquid-filled conduits, reducing NH_3_ flux and compromising selectivity [31]. This vulnerability is particularly pronounced for complex wastewater such as ADE, which contains natural organic matter, colloids, microorganisms, elevated ionic strength, and inorganic scaling precursors, including calcium, magnesium, carbonate, and phosphate species [32]. Such constituents can reduce effective surface tension, modify interfacial thermodynamics, and promote pore invasion, even under modest pressure differentials [33]. In addition, local pH gradients and concentration polarization near the membrane interface may induce supersaturation and precipitation of sparingly soluble inorganic minerals—such as calcium carbonate, calcium phosphate, and magnesium-based precipitates—during operation [34]. The accumulation of these inorganic deposits can partially block membrane pores, increase mass-transfer resistance, alter local surface wettability, and accelerate membrane wetting by disrupting the stability of the gas–liquid interface [35]. Furthermore, organic fouling, biofouling, and inorganic scaling may synergistically alter surface chemistry and create heterogeneous nucleation sites that facilitate wetting initiation and progressive pore flooding [36]. Thus, wetting, fouling, and scaling are strongly coupled interfacial phenomena that collectively undermine MC durability and long-term operational stability.

To mitigate membrane wetting, previous studies have developed and investigated superhydrophobic membrane architectures by combining low-surface-energy surface chemistry with hierarchical micro- and nanoscale roughness, thereby reducing liquid penetration into membrane pores and enhancing interfacial stability [37]. Biomimetic approaches inspired by lotus leaves, rose petals, or shark-skin textures have been implemented using silica nanoparticle deposition, electrospinning, plasma etching, sol–gel coatings, and fluorinated alkoxysilane grafting [[38], [39], [40], [41], [42]]. These strategies aim to increase liquid entry pressure (LEP), minimize solid–liquid contact fraction, and stabilize trapped air pockets, consistent with a Cassie–Baxter-type wetting regime [43]. However, static water contact angle (WCA) or the mere presence of an interfacial air layer does not inherently ensure wetting resistance under realistic wastewater conditions [44]. The fundamental barrier against liquid intrusion is the reverse Laplace pressure generated within the membrane pores, typically defined as the liquid entry pressure, Δ*P*_LEP_ = –2*γ*cos*θ*/*r*, where *γ* is the surface tension of the penetrating liquid, *θ* is the liquid contact angle on the membrane surface, and *r* is the effective membrane pore radius [45]. This implies that even if the interfacial air layer is destabilized, liquid entry can be prevented if the pore radius is sufficiently small and the local contact angle remains high enough to maintain a positive LEP. Pore-level stability depends on interfacial thermodynamics within tortuous membrane structures and on the persistence of air entrapment under high ionic strength and in organic-rich environments [46]. Complex matrices such as ADE can destabilize trapped air layers and induce transition toward a Wenzel-like state, characterized by increased solid–liquid contact and pore flooding [47]. Crucially, while an air layer effectively reduces the solid–liquid contact area to alleviate membrane fouling, the mechanisms and characterization requirements for fouling (surface accumulation) and wetting (pore-level intrusion) are significantly different [48]. Currently, membrane wetting is frequently inferred indirectly through flux decline or solute breakthrough, which limits the ability to resolve key issues regarding when pore invasion begins, how it propagates spatially, and how it progresses within the membrane structure [49].

Another challenge is that wetting is typically assessed *ex situ* or indirectly, obscuring the real-time structural evolution within membrane pores [50]. To bridge this gap, there is a critical need for *in situ* diagnostic tools capable of detecting whether and how much liquid has entered the membrane pores during operation. Optical coherence tomography (OCT), based on near-infrared backscattering, offers a non-destructive approach for the cross-sectional imaging of porous structures during operation [24]. When interpreted alongside performance metrics, operando OCT enables the visualization of wetting initiation, progression, and spatial heterogeneity, thereby supporting a mechanistic interpretation of interface stability [51]. In this context, a framework that directly links interfacial design, pore-level wetting dynamics, and NH_3_ transport performance under realistic ADE conditions remains lacking. Addressing this gap by transitioning from empirical surface modification toward rational interface engineering is essential for developing resilient superhydrophobic membrane contactors (SHMCs) capable of sustained performance in complex wastewater environments.

In this study, we developed and mechanistically validated a SHMC platform based on fluorinated-silane-engineered polyvinylidene fluoride (PVDF) membranes designed to stabilize a Cassie–Baxter-like gas–liquid interface for NH_3_ recovery from real ADE. The specific objectives of this study are to: (1) characterize the physicochemical and interfacial properties of the modified membranes; (2) quantify NH_3_ recovery performance, including recovery efficiency and membrane mass-transfer behavior; (3) elucidate the interfacial mechanisms governing pore-level wetting evolution and transport deterioration under chemically complex conditions; (4) employ operando OCT as a structural diagnostic tool to directly visualize pore flooding, fouling-associated structural evolution, and wetting-front propagation during operation; and (5) evaluate long-term durability through multi-cycle testing. Unlike conventional studies that infer wetting indirectly from flux decline or *ex situ* wettability measurements, this work establishes a direct mechanistic relationship between interfacial thermodynamics, pore-scale structural evolution, and NH_3_ transport behavior under realistic wastewater conditions. By integrating operando structural diagnostics with transport-performance analysis, this study advances SHMC research from empirical hydrophobic modification toward a mechanism-informed interfacial engineering framework for durable membrane contactor operation.

## 2 Materials and methods

### 2.1 Chemicals

For superhydrophobic membrane modification, ethanol (EtOH, 99.5%, reagent grade), benzyldimethyldodecylammonium chloride (DDBAC, 99%, reagent grade), (3-aminopropyl)triethoxysilane (APTES, 99%, reagent grade), acetic acid (High performance liquid chromatography, HPLC grade), and silicon dioxide nanoparticles (SiNPs, 10–20 nm, 99.5%) were purchased from Sigma-Aldrich (St. Louis, MO, USA). Fluorosilane reagents, including 1H,1H,2H,2H-perfluorooctyltriethoxysilane (PFOTES), 1H,1H,2H,2H-perfluorooctyltrimethoxysilane (PFOTMS), trimethoxy(1H,1H,2H,2H-heptadecafluorodecyl)silane (PFDTMS), and 1H,1H,2H,2H-perfluorodecyltriethoxysilane (PFDTES), were obtained from Gelest Inc. (Morrisville, PA, USA), GLPBIO technology LLC (Montclair, CA, USA), and Sigma-Aldrich, respectively, and used for the modification of superhydrophobic materials. Sulfuric acid (H_2_SO_4_, 99%) and sodium hydroxide (NaOH, 99%) were purchased from Sigma-Aldrich and used for pH adjustment of the feed and stripping solutions.

The real ADE used in this study was collected from the Chungju Food Bioenergy Center (Chungju, Republic of Korea); its detailed composition is provided in Supplementary Table S2.

### 2.2 Modifications of superhydrophobic membranes

(Fig. 1)

**Fig. 1:**
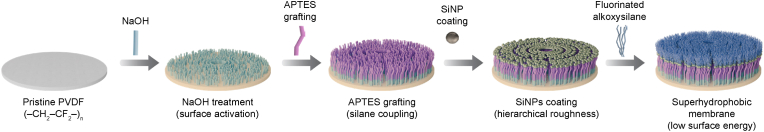
**Fabrication of superhydrophobic PVDF membranes**. Schematic of the sequential surface-modification procedure. Pristine poly(vinylidene fluoride) (PVDF) membranes were activated by NaOH treatment, functionalized with 3-aminopropyltriethoxysilane (APTES), coated with silica nanoparticles (SiNPs) to generate hierarchical surface roughness, and subsequently modified with fluorinated alkoxysilanes to reduce surface energy.

We employed a hydrophobic flat-sheet PVDF membrane (Durapore®, Merck Millipore, USA; nominal pore size, 0.45 μm; diameter, 47 mm) as the base substrate for the MC experiments. We designed a superhydrophobic modification strategy to construct a mechanically stable hierarchical roughness layer while simultaneously introducing low-surface-energy functional groups (Fig. 1). The modification protocol was adapted from previously reported methods, with additional steps incorporated to enhance functional-group grafting efficiency and improve the structural robustness of the hierarchical roughness layer [28]. Pristine PVDF membranes were first pre-wetted by immersion in EtOH until fully saturated to ensure uniform surface accessibility. The membrane surface was subsequently activated through treatment in an aqueous solution containing NaOH and DDBAC at 60 °C for 3 h. In this step, DDBAC acted as a cationic phase-transfer catalyst that facilitated the interaction of hydroxide ions with the hydrophobic PVDF surface, thereby promoting partial surface dehydrofluorination and increasing surface reactivity for subsequent silane grafting [52]. This activation step introduced reactive surface sites and improved the affinity of the membrane for subsequent silane grafting. To introduce amine-terminated functional groups to serve as anchoring sites for silica growth, the activated membranes were immersed in a mixture of APTES and EtOH at 35 °C for 1 h. During this step, amino-silane grafting occurred on the membrane surface while simultaneously initiating a sol-gel reaction. This process promoted the *in situ* formation of SiNPs, thereby generating a hierarchical micro/nanostructured roughness layer that is essential for achieving superhydrophobic surface characteristics. To further enhance particle coverage and improve the mechanical stability of the roughness layer, the membranes were subsequently dip-coated in an aqueous SiNP dispersion at 0.5% by weight (pH adjusted to 4.0 using acetic acid) at 35 °C for 3 h. This secondary coating step reinforced the hierarchical structure and increased the density of surface-bond nanoparticles. Finally, to reduce surface energy and stabilize the Cassie–Baxter interfacial state, the SiNP-coated membranes were fluorinated by immersion in EtOH containing 1% by volume fluorinated alkoxysilane (PFOTES, PFOTMS, PFDTES, or PFDTMS) for 30 min, followed by drying in a desiccator under ambient conditions. The resulting fluorinated membranes were denoted as PVDF@PFOTES, PVDF@PFOTMS, PVDF@PFDTES, and PVDF@PFDTMS, respectively.

The physicochemical properties of the fluorinated alkoxysilanes are summarized in Supplementary Table S3. These precursors were selected to investigate the combined effects of perfluoroalkyl chain length (C_8_ versus C_10_) and alkoxy-group reactivity (methoxy versus ethoxy). Crucially, the fluorine-to-carbon (F/C) ratios were recalculated based on the hydrolyzed state (Supplementary Table S3), as the alkoxy groups are removed during grafting. While PFOTES and PFOTMS (and similarly PFDTES and PFDTMS) result in identical F/C ratios after hydrolysis, their different reaction kinetics, driven by the steric effects of methoxy versus ethoxy groups, significantly influence the final grafting density and surface coverage. This systematic approach allows for a clearer interpretation of how molecular structure and silanization kinetics collectively dictate the resulting interfacial properties.

### 2.3 Characterization of superhydrophobic membranes

We used a suite of complementary analytical techniques to systematically characterize the physicochemical properties of the pristine and modified PVDF membranes. Fourier transform infrared (FTIR) spectroscopy was performed with an INVENIO S-type spectrometer (Bruker Optik GmbH, Germany) to identify surface functional groups and confirm chemical modifications of the PVDF membranes. The WCA, roll-off angle, and contact angle hysteresis (CAH) of the sample were measured using a contact angle analyzer (PHOENIX-300, Touch, SEO, Republic of Korea). A droplet (10 μL) of DI water was placed onto the membrane surface, and the image was taken side-on after 30 s. The roll-off angle ($\theta^{\prime }$) was measured at tilt angles from 1° to 90°. The CAH was calculated as the difference between the advancing angle and the receding angle. Furthermore, the apparent surface energy ($\gamma_{s}$) was determined using the Owens-Wendt-Rabel-Kaelble (OWRK) method [53]. The contact angles of two probing liquids, DI water (polar) and diiodomethane (dispersive), were measured to calculate the dispersive ($\gamma_{s,d}$) and polar ($\gamma_{s,p}$) components of the surface energy, according to the following equation (1):

(1) $$\gamma_{l}\left(1+\cos\;\theta \right)=2\sqrt{\gamma_{s,d}\gamma_{l,d}}+22\sqrt{\gamma_{s,p}\gamma_{l,p}}$$

where $\gamma_{l}$, $\gamma_{l,d}$, and $\gamma_{l,p}$ represent the total surface tension of the probing liquid and its dispersive and polar components, respectively. The total apparent surface energy is the sum of the two components ($\gamma_{s}$ = $\gamma_{s,d}$ + $\gamma_{s,p}$). This approach allowed for the quantification of the effective interfacial energy, considering the combined influence of chemical fluorination and hierarchical surface roughness. Surface chemical composition was analyzed by X-ray photoelectron spectroscopy (XPS) using a K-Alpha Plus spectrometer (Thermo Fisher Scientific, Waltham, MA, USA) equipped with an electron flood gun and a scanning ion source, with Al Kα radiation as the excitation source. Prior to XPS analysis, membrane samples were dried at 100 °C to remove adsorbed surface moisture. Surface morphology and elemental distribution were examined using a field-emission scanning electron microscope (FE-SEM; JSM-IT800, JEOL, Japan), coupled with energy-dispersive X-ray spectroscopy (EDX). Changes in surface topography and roughness induced by membrane modification were further quantified using three-dimensional laser scanning microscopy (3D-LSM; OLS5100, Olympus, Japan), enabling evaluation of micro- and nanoscale roughness features. Additionally, confocal laser scanning microscopy (CLSM; TCS SP8 HyVolution, Leica Microsystems, Germany) was employed to visualize the spatial distribution and infiltration depth of foulants. Riboflavin was utilized as a fluorescent model foulant, with excitation and emission wavelengths set at 488 and 500–600 nm, respectively, to distinguish between surface-adsorbed and matrix-embedded fouling layers.

### 2.4 Recovery of NH_3_ using SHMCs

#### 2.4.1 Experimental setup and operational conditions

(Fig. 2)

**Fig. 2:**
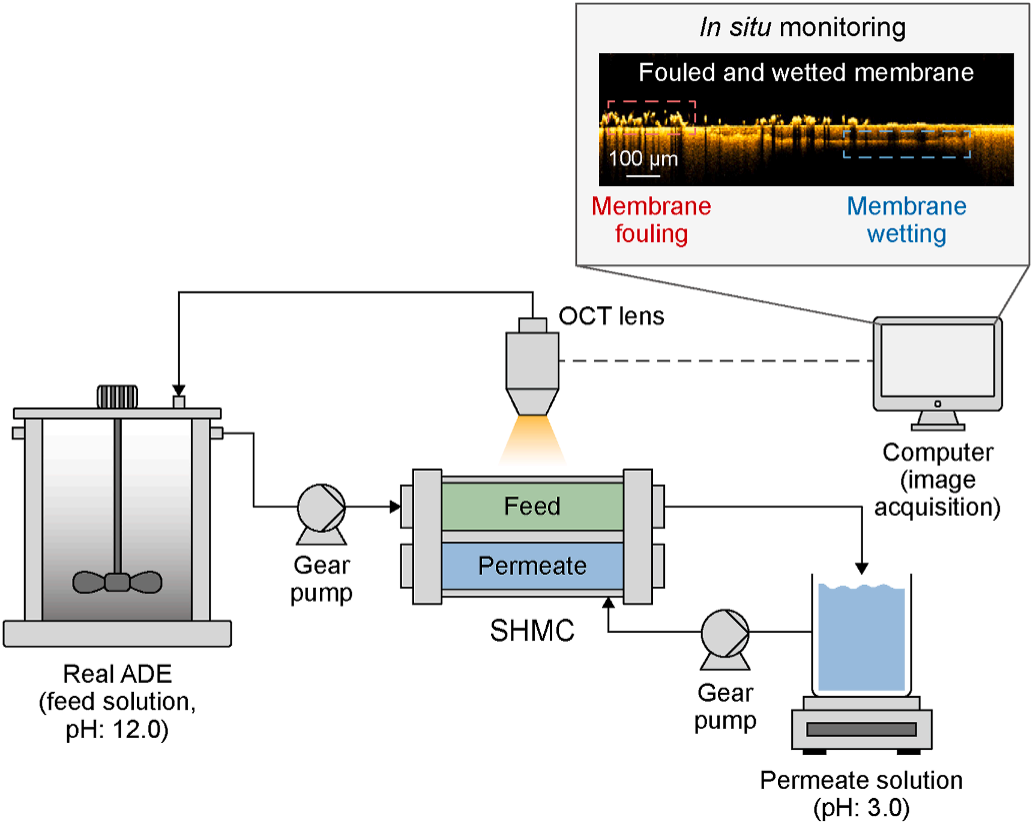
**Experimental setup for selective ammonia recovery and *in situ* membrane monitoring.** Schematic of the superhydrophobic membrane contactor (SHMC) system. The feed solution contained 250 mL of real anaerobic digestion effluent (ADE), adjusted to pH 12.0 using 1 mol L^−1^ NaOH to convert NH_4_^+^ to un-ionized NH_3_, whereas the stripping reservoir contained 250 mL of 0.5 mol L^−1^ H_2_SO_4_ at pH 3.0. The feed and stripping solutions were independently recirculated at 200 mL min^−1^ on opposite sides of the flat-sheet membrane using gear pumps, while the system temperature was maintained at 20 ± 1 °C. The effective membrane area was 13.85 cm^2^. NH_3_ transported through the membrane was protonated and retained as NH_4_^+^ in the acidic stripping solution. An optical coherence tomography probe was positioned above the membrane module to monitor the temporal evolution of membrane fouling and wetting *in situ*, and the acquired cross-sectional images were recorded and processed using a connected computer.

The SHMC-based system was designed to recover NH_3_ through transmembrane transport from the feed solution, followed by its capture in the stripping solution (Fig. 2; Supplementary Fig. S1). The modified PVDF membranes were mounted in a custom-fabricated acrylic module with an effective membrane area of 13.85 cm^2^. The NH_3_ recovery experiments were conducted under near-isothermal conditions without an intentional temperature gradient. Both the feed solution (250 mL of real ADE) and the stripping solution (250 mL of 0.5 mol L^−1^ H_2_SO_4_) were maintained at 20 ± 1 °C throughout the experiments and circulated at a constant flow rate of 200 mL min^−1^ using gear pumps (WT3000-1FA, Longer Pump Co., Ltd., China). The real ADE used as the feed solution was adjusted to pH 12.0 using 1 mol L^−1^ NaOH, thereby shifting the NH_4_^+^/NH_3_ equilibrium toward un-ionized NH_3_ and facilitating its transport across the membrane. The stripping solution was adjusted to pH 3.0 with 0.5 mol L^−1^ H_2_SO_4_, enabling selective capture of transported NH_3_ in the form of ammonium sulfate. Under these operating conditions, NH_3_ transport was governed primarily by the partial-pressure gradient of gaseous NH_3_ across the gas-filled hydrophobic membrane pores rather than by a thermally induced vapor-pressure gradient. Because the feed and stripping solutions were maintained at the same temperature, the water-vapor pressure difference across the membrane was minimal. Consequently, the volume of the stripping solution changed by less than 0.1% during operation, indicating negligible water transfer under the present conditions. Therefore, the high NH_3_ recovery performance mainly reflects selective NH_3_ gas transfer rather than water-vapor-driven membrane distillation.

The mass balance of the feed solution for NH_3_ was calculated using equation (2):

(2) $$V_{f}\frac{dC_{f,t}}{dt}=-KA\left(C_{f,t}-C_{p,t}\right)$$

where *V*_f_ is the volume of the feed solution (m^3^), *K* is the overall experimental mass transfer coefficient (m h^−1^), *A* is the effective membrane area (m^2^), *t* denotes the time (h), *C*_f,*t*_ is the NH_3_ concentration in the feed solution at time *t* (mg L^−1^), and *C*_p,*t*_ is the NH_3_ concentration in the stripping solution at time *t* (mg L^−1^). However, because the NH_3_ in the stripping solution is effectively nonvolatile at low pH values, *C*_p,*t*_ can be considered negligible. Therefore, the following modified equation (3) can be used:

(3) $$K=\ln\left(\frac{C_{f,0}}{C_{f,t}}\right)\frac{V_{f}}{At}$$

The mass flux of NH_3_ through the SHMC was determined using equation (4):

(4) $$J_{\text{FA}}=\frac{m_{s,t}-m_{s,0}}{At}$$

where *J*_FA_ represents the mass flux of NH_3_ through the SHMC (g m^−2^ d^−1^), *m*_s,*t*_ is the mass of NH_3_ in the stripping solution after the experiment (g), and *m*_s,0_ is the mass of NH_3_ in the stripping solution at the initial time (g).

#### 2.4.2 Analytical procedures

We used ion chromatography (IC) to determine the concentrations of nitrate nitrogen (NO_3_^−^-N) and nitrite nitrogen (NO_2_^−^-N) in the ADE. Anion analysis was performed using an IC system (LC-20ADSP, Shimadzu, Japan) equipped with an SI-90 4E column (4.0 × 250 mm, 9 μm; Shodex, Japan). The mobile phase consisted of 1.8 mM Na_2_CO_3_ and 1.7 mM NaHCO_3_, operated at a flow rate of 1.0 mL min^−1^ with the column oven maintained at 40 °C. Anions were detected using a conductivity detector (CDD-10AVP, Shimadzu, Japan). NH_3_, chemical oxygen demand (COD), and total phosphorus (TP) were quantified using commercial ammonium, COD, and TP test kits (HACH, USA) in accordance with the manufacturer's protocols. Total organic carbon (TOC) and total nitrogen (TN) concentrations were measured using a total organic carbon analyzer (TOC-L, Shimadzu, Japan).

### 2.5 Real-time monitoring of wetting and fouling in SHMCs

During SHMC operation, membrane wetting and fouling behavior were monitored *in situ* and in real time using OCT (LSM 03BB, Thorlabs, Germany). This noninvasive, high-resolution imaging technique enabled dynamic visualization of wetting propagation and foulant accumulation in both pristine PVDF membranes and superhydrophobic-modified membranes under operating conditions. The OCT system operates based on spectral-domain principles, with a central wavelength of 930 nm, and is equipped with a 5× telecentric objective lens, providing distortion-free surface and cross-sectional imaging. Time-resolved scans were acquired at a line rate of 30 kHz, allowing quantitative tracking of temporal evolution in interfacial and structural phenomena during SHMC operation.

We used cross-sectional OCT images to evaluate membrane structural features, including membrane layer thickness and pore-scale morphological changes associated with wetting or fouling. The wetting state was determined by analyzing the backscattered light intensity distribution, which is governed by the refractive index (RI) mismatch between the liquid, air, and the polymer matrix, with RIs of approximately 1.33, 1.0, and 1.42, respectively. A non-wetting state was identified by a sharp, high-intensity RI boundary at the liquid–membrane interface with a low-intensity (dark) region underneath, representing preserved air-filled pores. Conversely, wetting was identified by the infiltration of the liquid signal into these dark regions, leading to a loss of the air-filled porous contrast and a downward migration of the scattering interface [54]. We used ImageJ software (NIH, USA) to perform image processing and quantitative analysis. A consistent region of interest (ROI), defined as a fixed lateral segment of 500 μm encompassing the entire membrane thickness, was applied across all samples to ensure comparability and minimize sampling bias. Image binarization was conducted using the Otsu automatic thresholding algorithm to distinguish the polymer matrix (solid) region from the pore (void) region within the selected ROI. We calculated membrane porosity as the ratio of pore-region pixels to the total number of pixels within the ROI, following methods reported in previous studies [25]. For each membrane, porosity values were averaged over three representative cross-sectional slices. Given the inherent axial and lateral resolutions of the OCT system (2.0 and 3.0 μm, respectively), this analysis captures mesoscale structural changes rather than sub-micron pore features. Nevertheless, OCT provides robust real-time insight into pore-flooding behavior and fouling-associated structural evolution during operation. Overall, the integration of operando OCT enables quantitative real-time assessment of pore-level wetting dynamics and structural stability, thereby supporting the mechanistic interpretation of wetting resistance in SHMC-based separation processes.

## 3 Results and discussion

### 3.1 Characterization of modified superhydrophobic PVDF membranes

(Fig. 3)

**Fig. 3:**
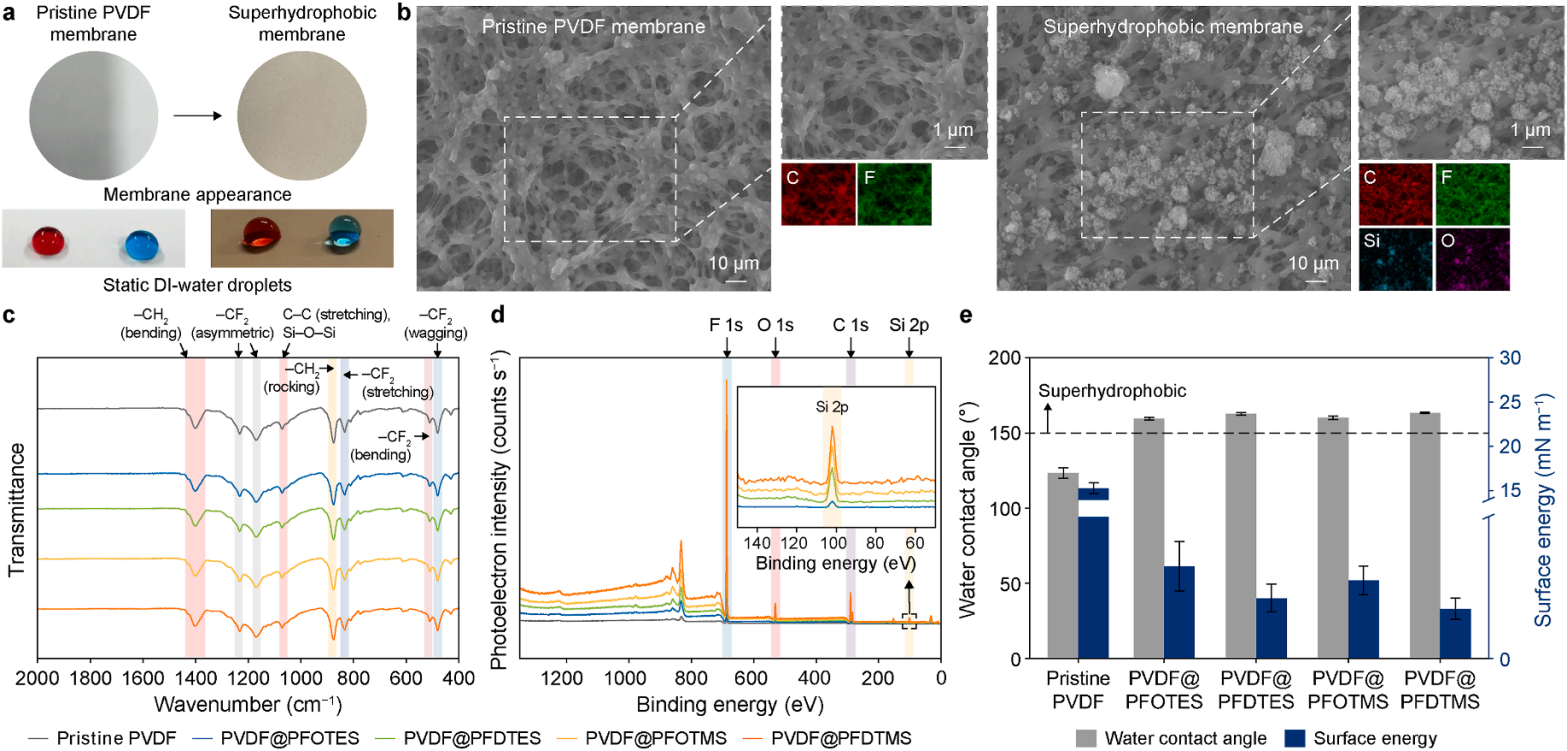
**Surface morphology and physicochemical properties of the modified PVDF membranes. a**, Photographs of pristine and superhydrophobic PVDF membranes and representative deionized (DI)-water droplets on their surfaces. **b**, Scanning electron microscopy images and corresponding energy-dispersive X-ray spectroscopy elemental maps of pristine PVDF and the superhydrophobic membrane. **c**, Fourier-transform infrared spectra of pristine PVDF and the four surface-modified membranes. **d**, X-ray photoelectron spectroscopy survey spectra; the inset shows the Si 2p region of the modified membranes. **e**, Water contact angle and apparent surface energy of the membranes. Data are presented as the mean ± standard deviation, and the error bars represent the standard deviation of independent measurements (*n* = 3).

The physicochemical characteristics of the superhydrophobic PVDF membranes were systematically evaluated following the stepwise surface modification process (Fig. 3). The pristine and modified PVDF membranes exhibited distinct macroscopic wetting behaviors, reflecting the progressive enhancement in surface hydrophobicity induced by the surface modification **(**Fig. 3a**)**. The pristine membrane exhibits limited water repellency with droplet spreading, whereas the modified membrane maintains nearly spherical droplets with minimal contact-area expansion. This pronounced contrast demonstrates a significant enhancement in apparent hydrophobicity following surface modification. Such behavior indicates the suppression of spontaneous imbibition and the formation of a solid–air–liquid composite interface, resulting from the combined effects of surface texture and fluorinated surface chemistry.

The pristine and modified membranes exhibited distinct surface morphologies and elemental distribution, as confined by SEM imaging and the corresponding EDS elemental mappings (Fig. 3b). The pristine PVDF membrane retains its intrinsic porous morphology and exhibits predominantly carbon (C) and fluorine (F) signals. In contrast, the modified membrane shows the incorporation of fine particulate SiNP aggregates discretely distributed over the porous substrate. Although the SiNPs do not form a completely continuous coating layer, they generate the hierarchical micro/nanoscale roughness necessary to stabilize the Cassie–Baxter-like state. The corresponding EDS elemental maps (detailed for each modification step a in Supplementary Fig. S2–S3) confirm the presence of silicon (Si) and oxygen (O) signals within the analyzed region, indicating that silica/siloxane species were incorporated into the modified surface layer. This discrete, non-continuous distribution of SiNPs is advantageous for SHMC applications because it provides the structural roughness required to enhance hydrophobicity while minimizing pore-blocking effects and preserving the effective surface area for mass transfer.

The chemical changes induced by each surface modification step were evaluated using the FTIR spectra of the pristine and modified PVDF membranes (Fig. 3c).The pristine PVDF membrane shows characteristic vibrational bands, including –CH_2_ bending at 1402 cm^−1^, –CF_2_ asymmetric stretching at 1171 and 1210 cm^−1^, and –CH_2_ rocking at 878 cm^−1^ [55]. The bands observed at 510 and 480 cm^−1^ are attributed to the –CF_2_ bending and –CF_2_ wagging vibrations, respectively [56]. Upon surface modification, the spectra exhibit key changes confirming successful chemical functionalization. The peak at 1070 cm^−1^, which corresponds to the C–C stretching of the polymer, shows a significant increase in intensity due to the overlap with the Si–O–Si stretching vibration [57]. This change indicates the formation of a siloxane network resulting from the hydrolysis and condensation of the silane precursors [58]. Furthermore, the intensification of the –CF_2_ stretching bands (near 1171–1210 cm^−1^) in the modified membranes reflects the successful attachment of fluorinated alkoxysilanes. These results, combined with the emergence of the Si–O–Si signal, provide strong evidence that the superhydrophobic coating was chemically integrated into the PVDF membrane rather than through simple physical deposition.

The surface chemical compositions of the pristine and modified membranes were further confirmed by XPS analysis, corroborating the successful incorporation of the targeted functional elements during each modification step (Fig. 3d). The pristine PVDF membrane is dominated by C 1s (290.2 eV) and F 1s (687.1 eV) signals corresponding to the PVDF polymer structure. After surface modification, distinct Si 2p (101.9 eV) and O 1s (531.2 eV) peaks emerge, directly confirming the presence of silane-derived and silica-related species at the membrane surface. Strong F 1s signals remained evident after fluorinated alkoxysilane treatment, confirming the successful incorporation and surface exposure of fluorine-rich functional groups. The simultaneous presence of Si- and O-containing species and fluorinated functionalities supports the proposed surface architecture, comprising a textured siloxane or silica-based scaffold functionalized with fluorinated terminal groups to reduce the surface energy.

The static WCA and calculated apparent surface energy were compared among the pristine and modified membranes to evaluate modification-induced changes in surface wettability (Fig. 3e). Pristine PVDF exhibits a relatively low WCA (123.3 ± 3.5°) and a higher surface energy (15.3 ± 0.6 mN m^−1^), reflecting insufficient water repellency. In contrast, all modified membranes exhibit WCAs exceeding the threshold for superhydrophobicity (WCA: 159.5–163.4°) and extremely low apparent surface energies (<0.3 mN m^−1^) (Supplementary Fig. S4). Notably, the surface energy values reported for the modified membranes represent the apparent surface energy (*γ*_s_), which is fundamentally modulated by the surface topography. While the intrinsic surface energy is a function of the fluorinated chemical moieties, the hierarchical roughness induced by SiNP deposition facilitates the transition to a Cassie–Baxter-like wetting state. In this regime, air pockets are trapped within the micro- and nanoscale asperities of the membrane surface. Since air possesses negligible surface energy, its entrapment effectively reduces the fractional solid–liquid contact area, leading to the observed ultra-low apparent surface energy. These results demonstrate that the combined introduction of hierarchical roughness and low-energy fluorinated chemistry effectively enhances interfacial water repellency by stabilizing a composite air–liquid interface. The variations in WCA and apparent surface energy among the modified membranes further suggest that differences in terminal-group chemistry and silane structure modulate the density of fluorinated groups and the stability of the trapped air layer, thereby influencing the final wetting performance.

### 3.2 Mechanistic insights into the stability of the Cassie–Baxter-like state

(Fig. 4)

**Fig. 4:**
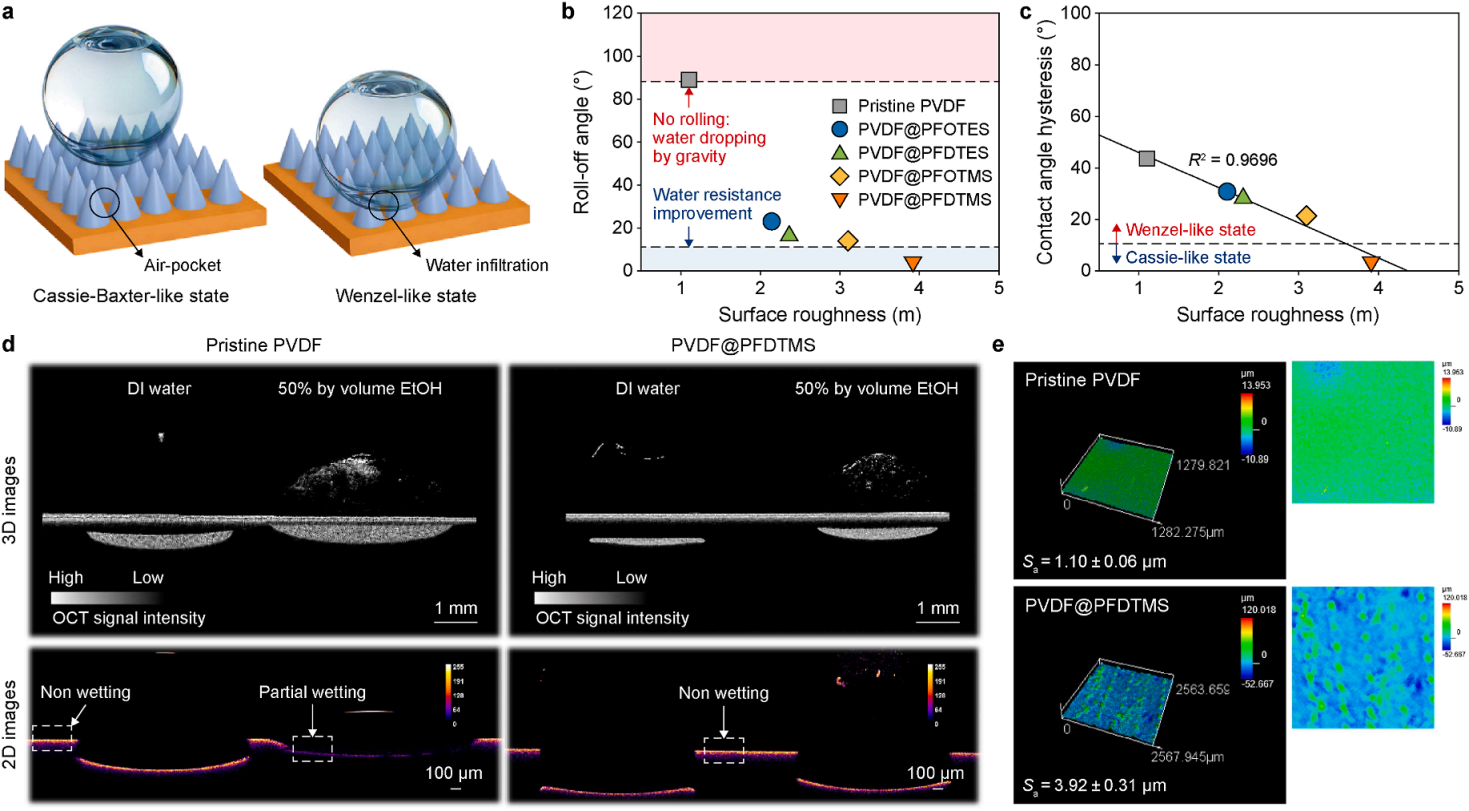
**Surface roughness and wetting states of the modified PVDF membranes. a**, Schematic illustration of Cassie–Baxter-like and Wenzel-like wetting states. **b**, Water-droplet roll-off angle as a function of areal surface roughness. The lower horizontal dotted line at 10° denotes the criterion for low droplet adhesion and enhanced water repellency, whereas the upper dotted line at 90° indicates that the droplet did not roll off before the membrane reached a vertical orientation and instead detached under gravity. **c**, Contact-angle hysteresis as a function of surface roughness; the line shows the linear fit. The horizontal dotted line at 10° denotes the operational threshold used to distinguish low-hysteresis Cassie-Baxter-like behavior below the line from higher-adhesion Wenzel-like behavior above the line. **d**, Three-dimensional and cross-sectional two-dimensional optical coherence tomography images of pristine PVDF and PVDF@PFDTMS after deposition of deionized (DI) water or 50% by volume ethanol. **e**, Three-dimensional laser-scanning microscopy topography and surface-height maps of pristine PVDF and PVDF@PFDTMS; the corresponding surface roughness (*S*_a_) values are indicated.

The surface modification enhanced the wetting stability of the PVDF membranes by combining fluorinated low-surface-energy chemistry with hierarchical micro- and nanoscale roughness, thereby shifting the apparent wetting state from Wenzel-like behavior toward a Cassie-Baxter-like regime (Fig. 4). A Cassie–Baxter-like state involves partial liquid–solid contact with air pockets retained beneath the droplet (Fig. 4a), reducing the effective liquid–solid contact fraction and droplet adhesion. In contrast, a Wenzel state is characterized by liquid impalement into surface asperities and pores, which increases the true solid–liquid contact area and strengthens three-phase contact-line pinning. This distinction is particularly important for porous membranes because Wenzel-like infiltration can initiate pore wetting and ultimately lead to breakthrough, whereas a stable Cassie-like configuration can delay liquid intrusion under capillary and hydrostatic driving forces [59].

The roll-off angle provides an application-relevant metric of water repellency because it quantifies the inclination required for gravity to overcome the droplet–surface adhesion associated with contact-line pinning. Pristine PVDF nearly exhibits non-shedding behavior (Fig. 4b), with a roll-off angle over 90° despite its relatively low surface roughness (*S*_a_ = 1.1 ± 0.1 μm), indicating strong pinning and substantial interfacial adhesion. Following surface modification, the roll-off angle decreases markedly across the fluorinated membrane series in parallel with increasing roughness, evidencing enhanced droplet mobility. PVDF@PFOTES (*S*_a_ = 2.1 ± 0.1 μm) shows a roll-off angle of 23.0 ± 0.8°, which further decreases to 16.3 ± 0.9° for PVDF@PFDTES (*S*_a_ = 2.4 ± 0.1 μm) and 14.0 ± 2.2° for PVDF@PFOTMS (*S*_a_ = 3.1 ± 0.1 μm). Notably, the roughest membrane, PVDF@PFDTMS (*S*_a_ = 3.9 ± 0.3 μm), achieves a roll-off angle of 4.3 ± 1.3°, corresponding to an overall reduction of approximately 95% relative to pristine PVDF. Collectively, these results indicate that hierarchical texturing, combined with fluorinated surface chemistry, substantially reduces droplet adhesion and promotes efficient droplet shedding, consistent with a progressive shift from an adhesion-dominated Wenzel-like (or mixed) regime on pristine PVDF toward a more Cassie-like regime on the modified membranes [60]. From an operational perspective, faster droplet removal shortens liquid residence time at the surface and reduces the likelihood of capillary entry into surface texture and membrane pores.

Consistent trends were observed for CAH, which directly reflects contact-line pinning. A strong inverse correlation was observed between surface roughness (*S*_*a*_) and CAH (*R*^*2*^ = *0.97*) across the membrane series (Fig. 4c). Pristine PVDF (*S*_a_ = 1.1 ± 0.1 μm) exhibits a high CAH of 43.5 ± 1.2°, consistent with strong adhesion and a Wenzel-like response. In contrast, PVDF@PFDTMS (*S*_a_ = 3.9 ± 0.3 μm) shows a much lower CAH of 3.6 ± 0.7°, approaching values typically associated with a Cassie–Baxter-like state. This pronounced reduction in CAH is attributed to the synergistic effect of physical texturing and chemical modification. Mechanistically, the hierarchical roughness facilitates air retention within the surface features, which drastically lowers the effective liquid–solid contact fraction. Simultaneously, the low surface energy imparted by the fluorinated silanes reduces the intrinsic molecular adhesion at the interface. The long-chain fluoroalkyl groups (e.g., in PFDTMS) create a chemically homogeneous, low-energy environment that minimizes the energy barrier for the receding contact line, thereby suppressing contact-line pinning and facilitating droplet motion. The slight variations in *S*_a_ among different silanes may stem from differences in the self-assembly and condensation behavior of the fluoroalkoxysilanes on the SiO_2_-decorated surface, where longer chains or different terminal structures slightly modulate the nano-structural packing density.

To directly assess wetting and infiltration on porous membranes, we used two- and three-dimensional OCT to image droplets on pristine PVDF and PVDF@PFDTMS under DI water and 50% by volume ethanol-water conditions (Fig. 4d). On pristine PVDF, OCT cross-sections in DI water already show features consistent with localized liquid intrusion into near-surface structures, while exposure to 50% by volume ethanol further enhances wetting. The reduced surface tension of the ethanol–water mixture lowers the capillary entry barrier, facilitating penetration into surface asperities and pores and yielding an enlarged basal contact region in the OCT images. In contrast, PVDF@PFDTMS maintains a comparatively elevated and discontinuous droplet base even under 50% by volume ethanol, indicating effective suppression of impalement into surface texture and membrane pores. This resistance under reduced surface-tension conditions supports a more stable Cassie-like configuration enabled by the combined effects of fluorinated chemistry and hierarchical roughness [61] (Supplementary Video S1).

The OCT observations are consistent with the surface morphologies measured by 3D-LSM (Fig. 4e). Pristine PVDF displays a relatively smooth topography with shallow asperities (*S*_a_ = 1.1 ± 0.1 μm), which can promote more continuous liquid–solid contact and facilitate local intrusion. In contrast, PVDF@PFDTMS exhibits pronounced hierarchical features with micro-scale protrusions and valleys (*S*_a_ = 3.9 ± 0.3 μm) (Supplementary Fig. S5). Such multiscale morphology is expected to favor air retention within surface cavities and reduce the effective liquid–solid contact fraction, thereby stabilizing a Cassie-like wetting configuration and improving wetting resistance, particularly under low-surface-tension conditions.

### 3.3 Recovery performance, wetting stability, and fouling behavior of SHMCs

(Fig. 5)

**Fig. 5:**
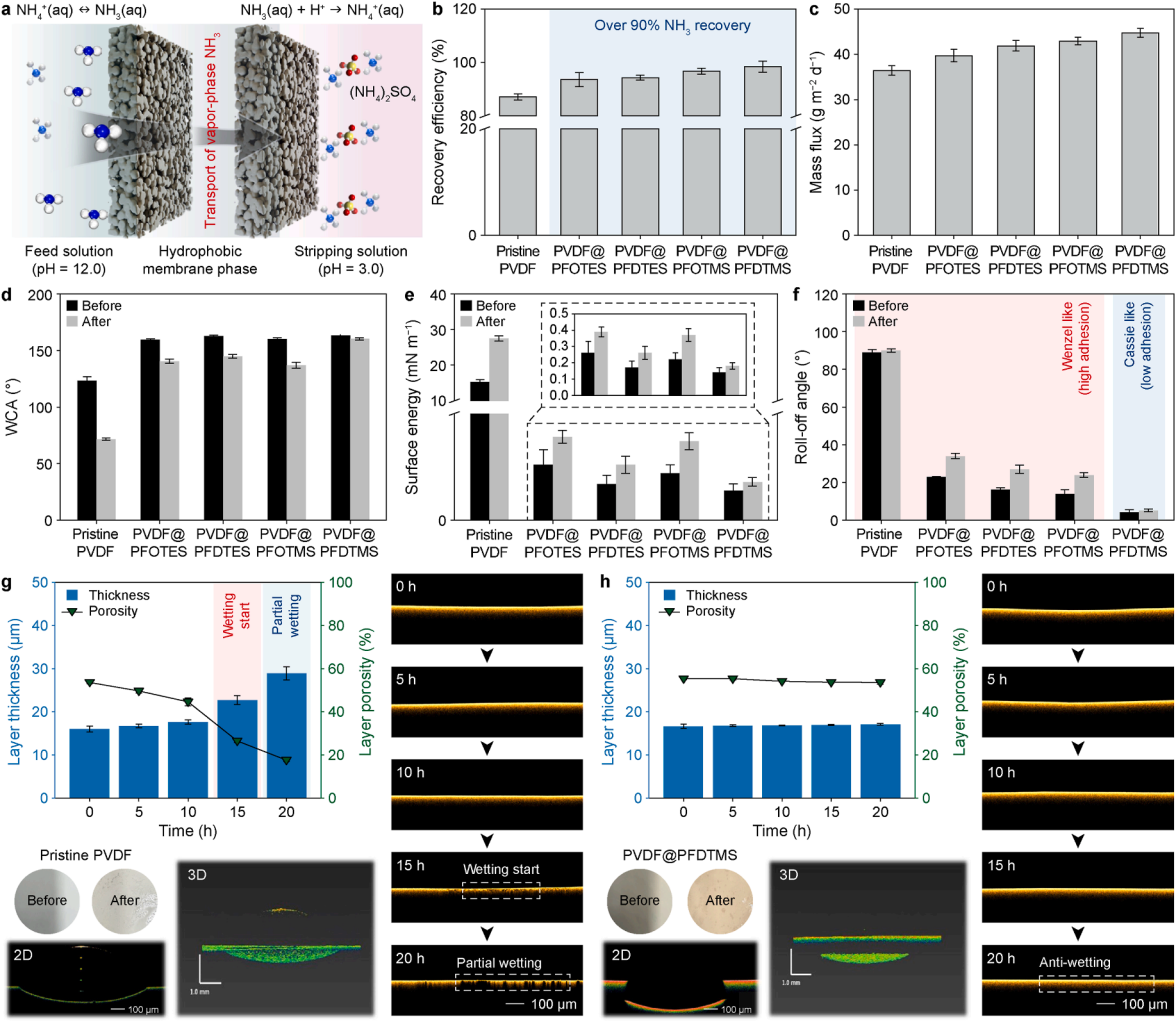
**Ammonia recovery and membrane wetting during SHMC operation. a**, Schematic of ammonia transport across the hydrophobic membrane. At high feed pH, NH_4_^+^ is converted to volatile NH_3_, which crosses the membrane and is protonated in the acidic stripping solution. **b**,**c**, Ammonia-recovery efficiency (**b**) and mass flux (**c**) of pristine PVDF and the four surface-modified membranes. **d**–**f**, Water contact angle (**d**), apparent surface energy (**e**), and roll-off angle (**f**) before and after operation. The inset in panel **e** shows an expanded view of the modified-membrane values. **g**,**h**, Temporal evolution of optical coherence tomography (OCT)-derived fouling-layer thickness and porosity for pristine PVDF (**g**) and PVDF@PFDTMS (**h**), together with representative membrane photographs and two- and three-dimensional OCT images. Data are presented as the mean ± standard deviation, and the error bars represent the standard deviation of independent measurements (n = 3).

The NH_3_ recovery mechanism, recovery performance, and interfacial wetting behavior in the SHMC were systematically evaluated using pristine and fluorinated PVDF membranes (Fig. 5). In parallel, membrane interfacial properties were characterized before and after 20 h of operation by combining real-time OCT imaging with measurements of static WCA, apparent surface energy, and roll-off angles. These complementary analyses were used to link macroscopic process performance to time-dependent wetting transitions and membrane structural evolution. The NH_3_ recovery mechanism in the SHMC involves the conversion of NH_4_^+^ to un-ionized NH_3_ in the alkaline feed, transmembrane diffusion through the gas-filled membrane pores, and subsequent protonation and capture as NH_4_^+^ in the acidic stripping solution (Fig. 5a). NH_3_ transport is primarily governed by pH-dependent speciation in the feed and diffusion through a non-wetted, air-filled membrane pore network. In the alkaline feed (pH = 12.0), the NH_4_^+^/NH_3_ equilibrium (p*K*_a_ = 9.25 at 25 °C) shifts toward molecular NH_3_, which is uncharged, volatile, and thus preferentially partitions into the gas phase within hydrophobic pores [15]. Maintaining a non-wetted pore structure is therefore essential for preserving a continuous gas pathway and minimizing mass-transfer resistance. Upon reaching the acidic stripping solution (pH = 3.0), NH_3_ is rapidly protonated to NH_4_^+^, effectively trapping nitrogen in the stripping phase and sustaining the transmembrane driving force by suppressing back-diffusion. When sulfuric acid is used as the stripping agent, the captured ammonium subsequently forms ammonium sulfate, (NH_4_)_2_SO_4_, enabling selective nitrogen recovery.

The NH_3_ recovery efficiency and mass flux were compared among membranes with different surface chemistries to evaluate the influence of surface modification on NH_3_ transport performance (Fig. 5b and c). Pristine PVDF delivered a recovery efficiency of 87.1 ± 1.2% with a mass flux of 36.5 ± 1.1 g m^−2^ d^−1^. This baseline performance is consistent with the favorable physicochemical characteristics of NH_3_ (low molecular weight, high volatility, and low hydration energy), which facilitate gas-phase partitioning and diffusion through hydrophobic polymeric pores. Fluorination led to a consistent improvement in both metrics: PVDF@PFOTES achieved 93.6 ± 2.6% and 39.7 ± 1.4 g m^−2^ d^−1^, while PVDF@PFDTES further increased to 94.3 ± 0.9% and 41.9 ± 1.2 g m^−2^ d^−1^. Notably, methoxy-terminated fluorosilanes (PVDF@PFOTMS and PVDF@PFDTMS) provided the highest performance, reaching 96.7 ± 1.1% and 98.4 ± 2.1%, with corresponding fluxes of 43.0 ± 0.8 and 44.8 ± 1.0 g m^−2^ d^−1^. Overall, the monotonic increase with the perfluoroalkyl chain length (and fluorine content) suggests that stronger hydrophobic/superhydrophobic character more effectively preserves non-wetted pores during operation, thereby maintaining an uninterrupted gas-phase transport pathway and lowering the operational mass-transfer resistance [28]. Among the tested membranes, PVDF@PFDTMS exhibited the best performance, indicating that the combination of hierarchical surface texture and ultra-low-energy chemistry provides robust interfacial conditions for sustained NH_3_ transport, particularly under conditions where partial pore wetting would otherwise limit mass transfer (Supplementary Fig. S6 and Table S4).

The evolution of the membrane wetting state during 20 h of SHMC operation was further quantified by changes in the WCA, apparent surface energy, and roll-off angle measured before and after operation, corroborating the observed differences in wetting stability among the membranes (Fig. 5d–f).Pristine PVDF underwent a pronounced wetting transition: the WCA decreased from 123.3 ± 3.5° to 71.6 ± 0.9° (41.9% reduction), while the surface energy increased from 15.3 ± 0.6 to 27.5 ± 0.7 mN m^−1^ (79.7% increase). These changes indicate a substantial loss of hydrophobicity and increased affinity toward polar species, plausibly driven by the adsorption of water and polar organic constituents in the feed and/or the formation of oxygen-containing surface functionalities. Consistently, the roll-off angle remained over 90°, implying strong contact-line pinning and a Wenzel-like wetting regime. In contrast, PVDF@PFDTMS exhibited excellent interfacial stability: the WCA decreased only slightly from 163.4 ± 0.5° to 160.3 ± 1.1°, and the roll-off angle remained very low (from 4.3 ± 1.3° to 5.2 ± 0.7°), consistent with a persistent Cassie-like state with weak droplet adhesion (Supplementary Fig. S7). The enhanced stability is attributed to outward-oriented perfluoroalkyl chains that create a low-energy surface and mitigate interactions with water and polar foulants, thereby suppressing liquid intrusion into the pore network (Supplementary Fig. S8 and Table S5–S6).

Real-time OCT provides direct evidence linking these interfacial changes to membrane structural evolution (Fig. 5g and h). To ensure spatial representativeness, imaging was performed at three distinct ROIs across the module, including the inlet, center, and outlet (see Supplementary Fig. S9 for additional cross-sections). Pristine PVDF exhibited pronounced and systemic structural degradation over 20 h (Fig. 5g), with the average thickness increasing from 16.0 ± 0.7 to 28.9 ± 1.5 μm (80.6% increase) and porosity decreasing from 53.6 ± 0.5 to 17.6 ± 0.6% (67.2% reduction). Wetting and fouling signatures emerged after approximately 15 h, followed by rapid performance deterioration. Correspondingly, OCT cross-sections evolved from relatively uniform scattering to increasingly heterogeneous intensity profiles. This included the development of broad low-intensity regions, indicating reduced optical contrast and increased attenuation consistent with liquid infiltration and pore wetting (potentially aided by refractive-index matching). While minor localized variations were observed, with slightly accelerated signal attenuation in downstream regions due to cumulative foulant deposition, the low coefficient of variation (<8.5%) across all ROIs confirms the spatial regularity of this wetting-induced collapse. The thickness increase in the pristine membrane is attributed to hydration-induced deformation, framework densification, and internal contaminant accumulation, consistent with wetting-induced collapse that has been reported previously [24]. While transient scattering from suspended particulates may contribute to short-lived fluctuations, the persistence and reproducibility of the OCT features, together with their agreement with ex-situ thickness/porosity measurements, indicate that the observed contrast evolution reflects genuine structural changes. By contrast, PVDF@PFDTMS maintained its structural integrity throughout the 20 h operation period (Fig. 5h). Only minor changes were observed (thickness: +3.0%, porosity: −3.3%), and OCT cross-sections remained uniformly bright and homogeneous across the membrane depth without the emergence of extended low-intensity regions. These results indicate that pore wetting and associated deformation were effectively suppressed by PFDTMS fluorination, thereby preserving the air-filled pore network and preventing the wetting-induced structural degradation observed for pristine PVDF.

To place the superior performance of PVDF@PFDTMS on a quantitative footing, the surface fluorine-to-carbon atomic ratio (F/C; Supplementary Table S7) was treated as a continuous descriptor of interfacial fluorination density. Across the series, the F/C ratio increased monotonically from pristine PVDF to PVDF@PFDTMS (0.506, 0.789, 0.856, 0.874, and 0.915 for pristine PVDF, PVDF@PFOTES, PVDF@PFDTES, PVDF@PFOTMS, and PVDF@PFDTMS, respectively). The 20 h NH_3_-recovery efficiency (*E*) increased linearly with F/C, following *E* = 25.6 (*F*/*C*) + 73.8 (*R*^2^ = 0.947; Spearman *ρ* = 1.00). The same descriptor governed the OCT-observed structural metrics: the porosity-retention rate increased, and the wetting-initiation time lengthened with F/C, from 32.8% retention and wetting at 15 h for pristine PVDF to 92.88% retention and no wetting within 20 h for PVDF@PFDTMS, with the intermediate membranes falling monotonically in between (Supplementary Fig. S10). The post-operation fluorine retention mirrored this trend, correlating with efficiency at an *R*^2^ of 0.984. A higher F/C ratio thus raises the areal density of –CF_2_/–CF_3_ moieties, lowers the surface free energy, and stabilizes the Cassie–Baxter-like state and LEP, simultaneously improving porosity retention, delaying wetting, and increasing recovery efficiency, identifying PVDF@PFDTMS as optimal because it maximizes this single interfacial descriptor.

### 3.4 Reusability performance with physical cleaning of SHMCs

(Fig. 6)

**Fig. 6:**
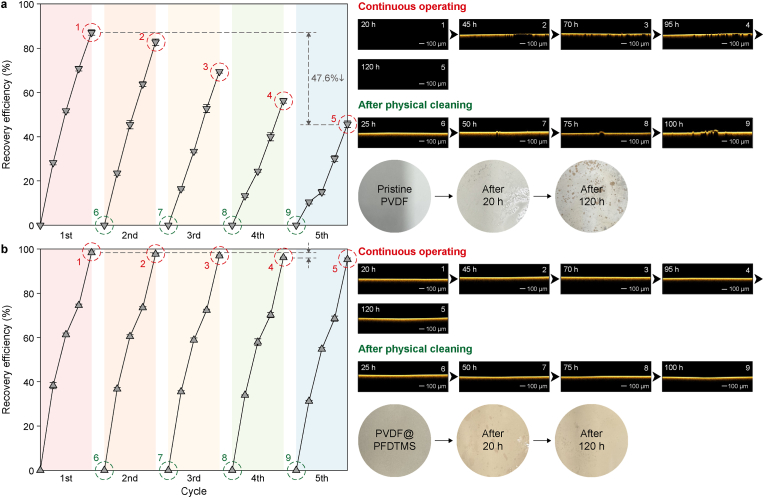
**Reusability and wetting evolution during repeated SHMC operation.** Ammonia-recovery efficiency and corresponding optical coherence tomography (OCT) observations for pristine PVDF (**a**) and PVDF@PFDTMS (**b**) over five operation–cleaning cycles. Red-circled points 1–5 correspond to OCT images collected during continuous operation at 20, 45, 70, 95, and 120 h, respectively. Green-circled points 6–9 correspond to OCT images collected after physical cleaning at 25, 50, 75, and 100 h, respectively. Membrane photographs show the samples before operation and after 20 and 120 h. The 47.6% value indicates the relative decrease in the final NH_3_ recovery efficiency of pristine PVDF from the first cycle to the fifth cycle. Data are presented as the mean ± standard deviation, and the error bars represent the standard deviation of independent experiments (*n* = 3).

The cyclic NH_3_ recovery performance of pristine PVDF and PVDF@PFDTMS was evaluated under repeated operation, with physical cleaning performed between cycles by DI water washing followed by drying (Fig. 6). In this study, we designed two steps to fulfill distinct roles: DI washing removes reversibly attached foulants via hydrodynamic rinsing, while drying is primarily used to restore the membrane's non-wetted state rather than as an additional foulant-removal step [33]. The recovery efficiency after this protocol reflects the extent of reversible fouling and the membrane's ability to re-establish its target interfacial condition between cycles. For pristine PVDF (Fig. 6a), the recovery efficiency decreases systematically with each cycle, resulting in a cumulative performance loss of 47.6% by the fifth cycle. Since DI washing is effective only for weakly bound deposits, this monotonic decline indicates the progressive accumulation of irreversible fouling that persists on and within the membrane. This fouling mechanism is further elucidated by CLSM analysis using riboflavin as a fluorescent model foulant (Supplementary Fig. S11). In the pristine PVDF, the fluorescent signals after cleaning exhibit a persistent, hazy distribution throughout the structure. This suggests that the foulants are not merely surface-adsorbed but are entrapped within the polymer matrix due to the high surface energy and subsequent pore wetting. Such matrix-embedded foulants are effectively shielded from the hydrodynamic shear forces during DI rinsing, leading to the observed decline in performance. The subsequent drying step failed to restore the initial performance, suggesting that the dominant limitation arose not from transient wetting but from residual foulant layers and pore blockage that continued to hinder membrane performance over successive cycles. This is consistent with time-resolved cross-sectional images, which show progressively pronounced interfacial features and non-uniform signals that remain even after cleaning, confirming the persistence of an adherent, irreversible layer facilitated by intimate liquid-solid contact.

In contrast, PVDF@PFDTMS (Fig. 6b) maintains consistently high recovery over five cycles, with negligible cumulative loss from 98.4 ± 2.1% to 95.3 ± 0.7%. This superior stability implies two favorable characteristics. First, foulants formed on PVDF@PFDTMS are predominantly reversible and easily removed by DI rinsing. The CLSM images (Supplementary Fig. S11) provide a compelling visual explanation for this: while the before cleaning state shows high fluorescence intensity, the signal is concentrated exclusively at the topmost interface. This indicates that the foulants remain suspended on the trapped air layer, characteristic of a Cassie–Baxter-like state, rather than infiltrating the porous matrix. Because the foulants are floating on this composite interface with minimal real contact area, they experience significantly reduced pinning forces. Consequently, these loosely attached deposits are readily removed by hydrodynamic shear during rinsing. Second, the drying step successfully restores the water-repellent state of the membrane, allowing it to re-establish a stable plastron prior to the subsequent cycle. Cross-sectional imaging supports this, showing limited interfacial deposit development and a profile that returns nearly to the baseline after cleaning. Mechanistically, this enhanced recoverability is attributed to the synergistic effect of low-surface-energy fluorinated chemistry and hierarchical roughness. This configuration maintains a stable air–liquid interface that suppresses persistent wetting and lowers foulant–surface adhesion. Furthermore, water droplets introduced during rinsing are less likely to infiltrate the texture; instead, they roll or slide across the superhydrophobic surface, mechanically entraining any loosely attached foulants. As a result, PVDF@PFDTMS effectively discourages the formation of compact, matrix-embedded fouling, favoring a regime where deposits remain weakly bound and highly reversible.

## 4 Implications for NH_3_ recovery from high-strength NH_3_ wastewater and long-term SHMC stability

The findings of this study provide important implications for the development of selective, durable, and energy-efficient SHMC systems for NH_3_ recovery from real ADE. Most notably, the PVDF@PFDTMS membrane achieved sustained high NH_3_ recovery by stabilizing a Cassie–Baxter-like gas–liquid interface under chemically complex conditions. Unlike pristine PVDF membrane, which exhibited rapid pore flooding and structural degradation, the superhydrophobic modification effectively suppressed liquid intrusion, preserved vapor transport pathways, and maintained high mass-transfer efficiency throughout multi-cycle operation. These results demonstrate that interfacial energy modulation, rather than static wettability alone, is central to sustaining NH_3_ selectivity and stable membrane performance. Beyond separation efficiency, robust NH_3_ recovery from ADE carries significant environmental and economic implications. From an environmental perspective, recovering NH_3_ as concentrated ammonium salts reduces nitrogen discharge to receiving waters, mitigating eutrophication risk while avoiding conversion to inert N_2_ through energy-intensive biological nitrification-denitrification pathways. This shift from removal to recovery decreases indirect greenhouse gas emissions associated with external carbon dosing, aeration energy, and sludge handling. Moreover, stabilizing NH_3_ in a recoverable form minimizes uncontrolled volatilization and associated atmospheric emissions. From an economic standpoint, selective NH_3_ capture enables valorization into industrial-grade ammonium products (e.g., ammonium sulfate), potentially generating recoverable value streams that offset operational costs (Supplementary Table S8–S9 and Text S1). Maintaining consistent vapor-phase transport under wetting-prone ADE conditions reduces process instability, minimizes unplanned membrane replacement, and lowers downtime-related expenses. In addition, improved wetting resistance decreases the likelihood of performance fluctuations that would otherwise increase chemical dosing variability in downstream absorption units. Collectively, these factors contribute to improved operational predictability and enhanced cost-efficiency of nutrient recovery systems.

Structural stability further distinguishes the engineered SHMC system. In contrast to pristine membranes that exhibited progressive pore flooding, increased effective thickness, and porosity collapse, the fluorinated membrane maintained structural integrity and near-invariant morphology during multi-cycle testing. Operando OCT imaging provided direct mechanistic evidence of sustained pore-level stability, confirming suppression of persistent liquid invasion and delayed fouling-induced wetting transitions. These observations underscore the importance of stabilizing the composite gas–liquid interface to prevent capillary intrusion and maintain consistent NH_3_ mass transfer under high ionic strength and organic-rich ADE matrices. Despite the promising performance demonstrated under controlled laboratory conditions, additional considerations are necessary for practical deployment. Real ADE introduces temporal variability in NH_3_ loading, elevated concentrations of dissolved salts, suspended solids, and diverse organic constituents that may influence wetting resistance, fouling behavior, and structural stability. Accordingly, future work will extend the present framework to continuous long-duration operation (exceeding one month) using real ADE to rigorously evaluate sustained performance beyond short-term laboratory cycles. Such extended evaluation will enable systematic assessment of time-dependent changes in recovery efficiency, wetting resistance, fouling kinetics, and structural integrity under realistic multi-component conditions. Furthermore, the long-term chemical stability of fluorinated silane modifications must be carefully evaluated. Potential leaching of perfluorinated functional groups under alkaline and high-ionic-strength conditions may affect membrane durability and environmental compatibility. Future investigations should therefore aim to quantify silane retention using surface-sensitive spectroscopic techniques, such as XPS and FTIR, establish a fluorine mass balance, and monitor the potential release of fluorinated species into the feed and stripping solutions during extended operation. Evaluating the stability of the low-surface-energy layer under repeated wetting-drying cycles and alkaline exposure will be critical for validating sustained applicability and ensuring regulatory compliance. By integrating mechanistic insight derived from operando structural imaging with environmental and economic performance considerations, extended validation using real wastewater, and chemical stability assessment of fluorinated interfaces, this work establishes a scientifically grounded foundation for rational SHMC design. Such a framework is essential for translating superhydrophobic membrane technologies from laboratory demonstration toward scalable, resilient, and environmentally responsible NH_3_ recovery systems in next-generation wastewater treatment. In addition, future work can also develop and validate a predictive-maintenance framework that regresses the early porosity-decay rate from operando OCT against long-term recovery efficiency to forecast optimal membrane-cleaning intervals.

## 5 Conclusions

In this study, we systematically evaluated NH_3_ recovery from NH_3_-rich ADE using SHMCs fabricated by modifying commercial PVDF membranes with superhydrophobic materials of different F/C ratios. Among the tested membranes, PVDF@PFDTMS exhibited the best performance, achieving the highest NH_3_ recovery with a recovery efficiency of 98.4 ± 2.1% and a mass flux of 44.8 ± 1.0 g m^−2^ d^−1^, outperforming pristine PVDF (87.1 ± 1.2%; 36.5 ± 1.1 g m^−2^ d^−1^). The enhanced performance of PVDF@PFDTMS is attributed to the synergistic effects of ultralow-surface-energy fluorinated chemistry and hierarchical surface roughness, which stabilize a Cassie–Baxter-like gas–liquid interface and preserve continuous air-filled transport pathways for NH_3_. Real-time OCT provided direct mechanistic evidence that wetting and fouling dynamics are strongly governed by membrane-surface design. Pristine PVDF underwent pronounced wetting/fouling-induced deterioration, reflected by a substantial loss of hydrophobicity (WCA decrease from 123.3 ± 3.5° to 71.6 ± 0.9°), a marked increase in surface energy (15.3 ± 0.6 to 27.5 ± 0.7 mN m^−1^), and severe structural collapse over 20 h (porosity: from 53.6 ± 0.5% to 17.6 ± 0.6%; thickness: from 16.0 ± 0.7 to 28.9 ± 1.5 μm). In contrast, PVDF@PFDTMS maintained high interfacial and structural stability, showing only minor changes (WCA: from 163.4 ± 0.5° to 160.3 ± 1.1°; thickness +3.0%; porosity −3.3%) and suppressing persistent pore flooding and interfacial deposit buildup. Overall, these results demonstrate that designing SHMCs around a Cassie–Baxter-like, self-cleaning interface, validated by operando OCT, is an effective strategy to mitigate the coupled wetting-fouling failure modes that limit conventional MCs. The proposed PVDF@PFDTMS-based SHMC platform therefore provides a robust and energy-efficient route for durable NH_3_ recovery from real ADE, with clear potential for scalable nutrient recovery applications under realistic wastewater conditions.

## CRediT authorship contribution statement

**Hongrae Im:** Writing – original draft, Visualization, Methodology, Formal analysis, Data curation, Conceptualization. **Am Jang:** Writing – review & editing, Validation, Supervision, Methodology, Funding acquisition, Formal analysis, Conceptualization.

## Declaration of competing interest

The authors declare that they have no known competing financial interests or personal relationships that could have appeared to influence the work reported in this paper.
