# Supplementary material for: Durable superhydrophobic interfaces sustain long-term ammonia recovery from real anaerobic digestion effluent
Source: Environ Sci Ecotechnol. 2026 Aug 4;33:100748. doi: 10.1016/j.ese.2026.100748 (PMC13463251; doi:10.1016/j.ese.2026.100748)
Supplement: Supplementary file 1 [file mmc1.docx]

*Supplementary material*

**Durable superhydrophobic interfaces sustain long-term ammonia recovery from real anerobic digestion effluent**

Hongrae Im and Am Jang^*^

Department of Global Smart City, Sungkyunkwan University (SKKU), 2066, Seobu-ro, Jangan-gu, Suwon, Gyeonggi-do 16419, Republic of Korea

^*^Corresponding author email: amjang@skku.edu (A. Jang)

E-mail address: [ky421100@naver.com](mailto:ky421100@naver.com) (H. R. Im)

Target journal: Environmental Science and Ecotechnology

Number of pages: 27

Figures: S1-S11

Tables: S1-S9

Text: S1

**Table S1.** Comparison of various ammonia recovery technologies from wastewater

| **Technology** | **Wastewater type** | **Key advantages** | **Limitations** | **Ref.** |
| --- | --- | --- | --- | --- |
| Adsorption | Real swine wastewater  (NH_4_^+^: 400 mg L^-1^) | - High selectivity for NH_4_^+^ using zeolites - Simple operation - Potential for direct use as soil conditioner | - Competitive adsorption by cations (K^+^, Na^+^) - Frequent chemical regeneration - Pore clogging by organics/solids | [1] |
| Air or steam stripping | Anesthetic gas plant wastewater  (NH_4_^+^: 16,240 mg L^-1^) | - Effective for high-strength wastewater  (> 2,000 mg L^-1^) - Simple and robust process | - High chemical cost for pH adjustment (pH > 11) - Performance drops at low temperature | [2] |
| Ion exchange | Synthetic ammonia wastewater  (NH_4_^+^: 100 mg L^-1^) | - Significantly higher capacity and faster kinetics than raw zeolites - Synergistic ion exchange and chemical reaction | - High synthesis cost - Susceptibility to nZVI oxidation (passivation) - Potential leaching of nanoparticles | [3] |
| Electrolytic precipitation  (Struvite) | Anaerobically digested poultry wastewater  (NH_4_^+^: 456.5 mg L^-1^) | - High-purity fertilizer recovery - Reduced chemical dependency via electrochemical pH control - Effective for complex poultry wastewater | - High electricity consumption - Electrode passivation and fouling - Complex system maintenance and organic acid costs | [4] |
| Bipolar membrane electrodialysis  (BMED) | Anaerobic digestion  (NH_4_^+^: 700 ± 170 mg L^-1^) | - In-situ acid/base generation without external chemicals - Pulse modulation reduces energy and fouling | - Very high membrane cost - Complex electrical control systems - Risk of membrane delamination | [5] |
| Submerged ultrafiltration (UF) with air stripping | Aqueous phase obtained from supercritical, hydrothermal processing of sewage sludge  (NH_4_^+^: 6,700 mg L^-1^) | - Effective for oily and high particle wastewater (e.g., HTL) - High recovery rate (> 90%) - Simultaneous oil and nutrient recovery | - Rapid UF membrane fouling - High energy for air supply and heating - Large amounts of chemical for pH adjustment | [6] |
| Microfiltration (MF) + vacuum evaporation  (Ceramic membrane) | Raw pig manure slurry  (NH_4_^+^: 2,200 mg L^-1^) | - Produces high-purity ammonia-water - Simultaneous concentration of N, P and K - Suitable for high-strength pig manure | - Extremely high energy demand for evaporation - Severe MF membrane fouling - High capital and maintenance costs | [7] |
| Membrane contactor (MC) | Anaerobic digester centrate  (NH_4_^+^: 1,814 mg L^-1^) | - High selectivity of NH_3_ gas - Compact footprint - High specific interfacial area - Direct production of liquid fertilizers - Low hydraulic energy demand | - Vulnerable to membrane wetting by surfactants or organics | [8] |

**Table S2**. The component of real ADE

| **Parameters** | **Value** | **Unit** |
| --- | --- | --- |
| pH | 7.6 | - |
| TOC | 780.6 ± 14.2 | mg L^-1^ |
| tCODcr | 1,484.5 ± 21.6 |  |
| TN | 2,744.5 ± 16.7 |  |
| NH_3_-N | 2,447.1 ± 5.9 |  |
| NO_3_-N | 6.4 ± 0.5 |  |
| NO_2_-N | 3.1 ± 1.1 |  |
| TP | 57.4 ± 1.8 |  |
| SS | 3,024.2 ± 54.5 |  |
| VSS | 430.6 ± 17.1 |  |

**Table S3**. Physicochemical properties of the grafted moieties of fluorinating agents

| **Superhydrophobic material** | **Chemical formula** | **Grafted moiety (After hydrolysis)** | **Fluorine-to-carbon ratio (F/C)** | **Molecular weight  (g/mol)** |
| --- | --- | --- | --- | --- |
| PFOTES | C_14_H_19_F_13_O_3_Si | C_8_F_13_H_4_Si- | 1.63 | 510.4 |
| PFOTMS | C_11_H_13_F_13_O_3_Si | C_8_F_13_H_4_Si- |  | 468.3 |
| PFDTES | C_16_H_19_F_17_O_3_Si | C_10_F_17_H_4_Si- | 1.70 | 610.4 |
| PFDTMS | C_13_H_13_F_17_O_3_Si | C_10_F_17_H_4_Si- |  | 568.3 |

**Fig. S1**. Flowchart of real-time monitoring of wettability and recovery of NH_3_ using SHMC process

**Fig. S2.** SEM images and EDS elemental mapping of the PVDF membrane modification procedure

**Fig. S3**. SEM images with EDS mapping of modified PVDF membranes

**Fig. S4.** Water droplets on modified PVDF membrane surfaces

**Fig. S5**. 3D-LSM images of modified PVDF membranes

**Fig. S6.** Experimental overall mass transfer coefficient of modified PVDF membranes

**Table S4**. Overall mass transfer coefficients of NH_3_ on pristine and superhydrophobic-modified PVDF membranes

| **Resource** | **Overall mass transfer coefficient (m h^-1^)** | | | | |
| --- | --- | --- | --- | --- | --- |
|  | **Membrane configuration** | | | | |
|  | **Pristine**  **PVDF** | **PVDF@ PFOTES** | **PVDF@ PFDTES** | **PVDF@ PFOTMS** | **PVDF@ PFDTMS** |
| **NH_3_** | 3.21 × 10^-2^ | 4.09 × 10^-2^ | 4.48 × 10^-2^ | 5.06 × 10^-2^ | 6.23 × 10^-2^ |

**Fig. S7.** Surface roughness of modified PVDF membranes before and after 20 h operation

**Fig. S8**. SEM images of modified PVDF membranes before and after 20 h operation

**Table S5**. SEM-EDS of pristine and superhydrophobic-modified PVDF membranes before operation

| **Element** | **Membrane configuration** | | | | |
| --- | --- | --- | --- | --- | --- |
|  | **Pristine PVDF** | **PVDF@ PFOTES** | **PVDF@ PFDTES** | **PVDF@ PFOTMS** | **PVDF@ PFDTMS** |
| C | 66.40 | 53.74 | 50.87 | 49.18 | 50.01 |
| F | 33.60 | 42.38 | 43.56 | 42.98 | 45.74 |
| O | - | 3.15 | 4.46 | 6.21 | 3.36 |
| Si | - | 0.73 | 1.11 | 1.63 | 0.89 |
| Total | 100 | 100 | 100 | 100 | 100 |

**Table S6**. SEM-EDS of pristine and superhydrophobic-modified PVDF membranes after operation

| **Element** | **Membrane configuration** | | | | |
| --- | --- | --- | --- | --- | --- |
|  | **Pristine PVDF** | **PVDF@ PFOTES** | **PVDF@ PFDTES** | **PVDF@ PFOTMS** | **PVDF@ PFDTMS** |
| C | 73.62 | 56.50 | 53.68 | 52.52 | 51.10 |
| F | 21.74 | 37.64 | 40.15 | 38.69 | 44.24 |
| O | 3.61 | 4.36 | 4.92 | 6.74 | 3.67 |
| Si | - | 0.62 | 0.78 | 1.45 | 0.82 |
| N | 0.74 | 0.67 | 0.32 | 0.48 | 0.11 |
| P | 0.29 | 0.21 | 0.15 | 0.12 | 0.06 |
| Total | 100 | 100 | 100 | 100 | 100 |

**Fig. S9.** OCT cross-section images of **(a)** pristine PVDF and **(b)** PVDF@PFDTMS at the left (inlet), center, and right (outlet) positions

**Table S7.** Comparison of membranes on different quantitative correlation

| **Membrane** | **F/C (EDS)** | **Eff. 20h (%)** | **Porosity ret. (%)** | **Wetting (h)** | **F-loss 20h (%)** |
| --- | --- | --- | --- | --- | --- |
| Pristine | 0.506 | 87.1 | 32.77 | 15 | 11.86 |
| PVDF@PFOTES | 0.789 | 93.6 | 90.75 | > 20 | 4.74 |
| PVDF@PFDTES | 0.856 | 94.3 | 90.32 | > 20 | 3.41 |
| PVDF@PFOTMS | 0.874 | 96.7 | 91.85 | > 20 | 4.29 |
| PVDF@PFDTMS | 0.915 | 98.4 | 92.88 | > 20 | 1.50 |

**Fig. S10.** OCT images of PVDF@PFOTES, PVDF@PFOTMS, and PVDF@PFDTES membranes after long-term operation.

**Fig. S11.** CLSM images of pristine PVDF and PVDF@PFDTMS membranes of before and after cleaning

**Table S8**. Price of the reagents for membrane modification (per m^2^).

| **Reagent** | **Loading/m^2^** | **$/m^2^ single** | **Share** | **reused ×15** | **Unit price** |
| --- | --- | --- | --- | --- | --- |
| Fluorosilane (PFDTMS) | 1 vol% bath, 16 g | 48.00 | 76% | 3.20 | $3/g |
| APTES | 2 vol% bath, 19 g | 12.49 | 20% | 0.83 | $0.66/g |
| Silica NPs | 0.5 wt% bath, 5 g | 0.50 | 1% | 0.03 | $0.10/g |
| Ethanol | 2 L pre-wet+solvent | 2.00 | 3% | 0.13 | $1/L |
| DDBAC + NaOH | activation bath, 1 g | 0.01 | 0% | 0.00 | $0.015/g |
| TOTAL modifiers | - | 63 | 100% | 4.2 | - |

**Table S9.** Price of the recovered nitrogen and show payback.

| **Quantity** | **Value** | **Basis** |
| --- | --- | --- |
| N recovered | 14.8 kg N/m^2^/yr | 44.8 g/m^2^/d × 330 d |
| Ammonium sulfate (AS) | 69.7 kg AS/m^2^/yr | ×4.72 kg AS per kg N |
| AS sales value | $20.9 /m^2^/yr | $300/ton |
| + Avoided N-removal | $44.4 /m^2^/yr | $3/kg N |
| Combined value | $65.3 /m^2^/yr |  |
| Payback (modifiers) | 2.4 months (AS only) | vs $4.2/m^2^ reused |
| Payback (full membrane) | 1.4 years | $94/m^2^ ÷ combined |

**Text S1. Economic feasibility of superhydrophobic modification for NH_3_ recovery from ADE**

From an economic standpoint, the superhydrophobic modification adds only a modest increment to the membrane cost. The modifiers are applied from dilute, reusable baths, so the consumed quantities are small; DDBAC, in particular, is a low-cost commodity biocide and is not a cost driver, while the fluoroalkylsilane accounts for roughly three-quarters of the incremental reagent cost. On a conservative single-use basis the modification adds $64/m^2^, falling to $4/m^2^ with bath reuse, small relative to the commercial PVDF substrate ($50-100/m^2^ at industrial scale) (**Table S8**). On the revenue side, at a mass flux of 44.8 g/m^2^/d each square metre recovers 14.8 kg-N/yr (70 kg (NH_4_)_2_SO_4_ per year, $21/m^2^/yr at $300/t), rising to $65 m^2^/yr when the avoided cost of conventional biological nitrogen removal ($3/kg-N) is credited (**Table S9**). The incremental modification cost is thus offset within months, and the anti-wetting stability further extends membrane lifespan and lowers cleaning and replacement frequency, reducing lifecycle cost relative to unmodified PVDF. Because the ADE wastewater can vary significantly depending on locations, feed type, seasonal variations, plant size, and so on, refined process designs based on the compositions and characteristics of ADE could also be important to further improve the system efficiency.

**References**

[1] A. Damodara Kannan, P. Parameswaran, Ammonia adsorption and recovery from swine wastewater permeate using naturally occurring clinoptilolite, Journal of Water Process Engineering 43 (2021) 102234.

[2] F. Ulu, M. Kobya, Ammonia removal from wastewater by air stripping and recovery struvite and calcium sulphate precipitations from anesthetic gases manufacturing wastewater, Journal of Water Process Engineering 38 (2020) 101641.

[3] O. Eljamal, R. Eljamal, I. Maamoun, A.M.E. Khalil, T. Shubair, O. Falyouna, Y. Sugihara, Efficient treatment of ammonia-nitrogen contaminated waters by nano zero-valent iron/zeolite composite, Chemosphere 287 (2022) 131990.

[4] R.J.N. Aka, M.M. Hossain, A. Nasir, Y. Zhan, X. Zhang, J. Zhu, Z.-W. Wang, S. Wu, Enhanced nutrient recovery from anaerobically digested poultry wastewater through struvite precipitation by organic acid pre-treatment and seeding in a bubble column electrolytic reactor, Water Research 252 (2024) 121239.

[5] I. Kaniadakis, J.B. van Lier, H. Spanjers, Ammonia recovery in bipolar membrane electrodialysis via pH control through electric current pulse modulation, Water Research 286 (2025) 124185.

[6] A. Sayegh, N. Shylaja Prakash, T.H. Pedersen, H. Horn, F. Saravia, Treatment of hydrothermal liquefaction wastewater with ultrafiltration and air stripping for oil and particle removal and ammonia recovery, Journal of Water Process Engineering 44 (2021) 102427.

[7] P. Samanta, L. von Ungern-Sternberg Schwark, H. Horn, F. Saravia, Nutrient recovery and ammonia-water production by MF-vacuum evaporation treatment of pig manure, Journal of Environmental Chemical Engineering 10(1) (2022) 106929.

[8] A.M. Schwiebert, J.A. Bush, C. Bellona, J. Vanneste, T.Y. Cath, Membrane Contactors for Ammonia Recovery from Anaerobic Digester Centrate: Pretreatment and Process Optimization, ACS ES&T Water 4(8) (2024) 3284-3296.
